# Direct Evidence for Packaging Signal-Mediated Assembly of Bacteriophage MS2

**DOI:** 10.1016/j.jmb.2015.11.014

**Published:** 2016-01-29

**Authors:** Óttar Rolfsson, Stefani Middleton, Iain W. Manfield, Simon J. White, Baochang Fan, Robert Vaughan, Neil A. Ranson, Eric Dykeman, Reidun Twarock, James Ford, C. Cheng Kao, Peter G. Stockley

**Affiliations:** 1 Astbury Centre for Structural Molecular Biology, University of Leeds, Leeds LS2 9JT, United Kingdom; 2 Department of Molecular and Cellular Biochemistry, Indiana University, Bloomington, IN 47405, USA; 3 The Center for Genomics and Bioinformatics, Indiana University, Bloomington, IN 47405, USA; 4 Department of Biology and Mathematics and York Centre for Complex Systems Analysis, University of York, York YO10 5DD, United Kingdom

**Keywords:** CP, coat protein, MP, maturation protein, PS, packaging signal, ssRNA, single-stranded RNA, EM, electron microscopy, EDTA, ethylenediaminetetraacetic acid, RNA virus, assembly mechanism, RNA–protein interaction, packaging signals

## Abstract

Using cross-linking coupled to matrix-assisted laser desorption/ionization mass spectrometry and CLIP-Seq sequencing, we determined the peptide and oligonucleotide sequences at the interfaces between the capsid proteins and the genomic RNA of bacteriophage MS2. The results suggest that the same coat protein (CP)–RNA and maturation protein (MP)–RNA interfaces are used in every viral particle. The portions of the viral RNA in contact with CP subunits span the genome, consistent with a large number of discrete and similar contacts within each particle. Many of these sites match previous predictions of the locations of multiple, dispersed and degenerate RNA sites with cognate CP affinity termed packaging signals (PSs). Chemical RNA footprinting was used to compare the secondary structures of protein-free genomic fragments and the RNA in the virion. Some PSs are partially present in protein-free RNA but others would need to refold from their dominant solution conformations to form the contacts identified in the virion. The RNA-binding peptides within the MP map to two sections of the N-terminal half of the protein. Comparison of MP sequences from related phages suggests a similar arrangement of RNA-binding sites, although these N-terminal regions have only limited sequence conservation. In contrast, the sequences of the C-termini are highly conserved, consistent with them encompassing pilin-binding domains required for initial contact with host cells. These results provide independent and unambiguous support for the assembly of MS2 virions via a PS-mediated mechanism involving a series of induced-fit viral protein interactions with RNA.

## Highlights

• CLIP-Seq reveals a defined set of RNA–coat interactions in a single-stranded RNA virion.
• RNA sites match previous predictions of multiple PSs.
• Data provide unambiguous confirmation of PS-mediated assembly.

## Graphical abstract

**Figure:**
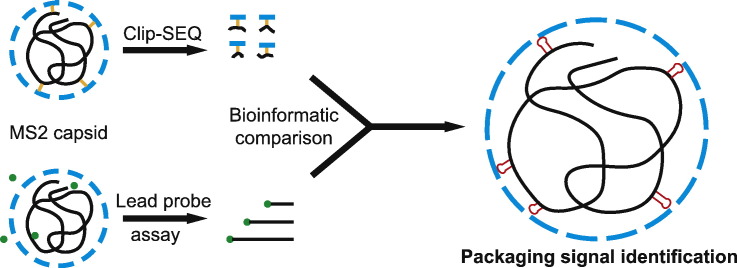

## Introduction

Spherical viral capsids assemble by two distinct mechanisms [1]. Many double-stranded DNA phages and Herpesviruses form a protein-only shell of coat and scaffold proteins, a pro-capsid, into which the genome is actively pumped via specialzsed energy-requiring enzymes as the scaffold proteins are removed. Spherical, positive-sense, single-stranded RNA (ssRNA) viruses, which include many major pathogens in all kingdoms of life, assemble by the co-assembly of coat protein (CP) subunits around the genome [2]. The free energy for this process has been thought to come mostly from favorable electrostatic interactions between the negatively charged RNA and positively charged residues and domains in the CPs [3], [4], [5], [6], [7], [8], [9], [10], [11], [12]. Adding to this view are the observations from *in vitro* reassembly experiments showing that many viral CPs can assemble into virus-like particles in the absence of RNA, in the presence of non-cognate RNAs, or even anionic polymers. High-resolution X-ray structures of ssRNA viruses mostly reveal only a limited ordering of the encapsidated genome, again implying that RNA has no, or very little, overt roles in virion assembly [13], [14], [15], [16], [17]. However, the specificity of genome packaging is incompletely explained by such an assembly mechanism. Virions from natural infections predominantly encapsidate their cognate genomes, with only minor misincorporation of cellular or degraded RNAs [18], [19]. These outcomes hint at more complex regulation of the assembly process.

**Fig. 1:**
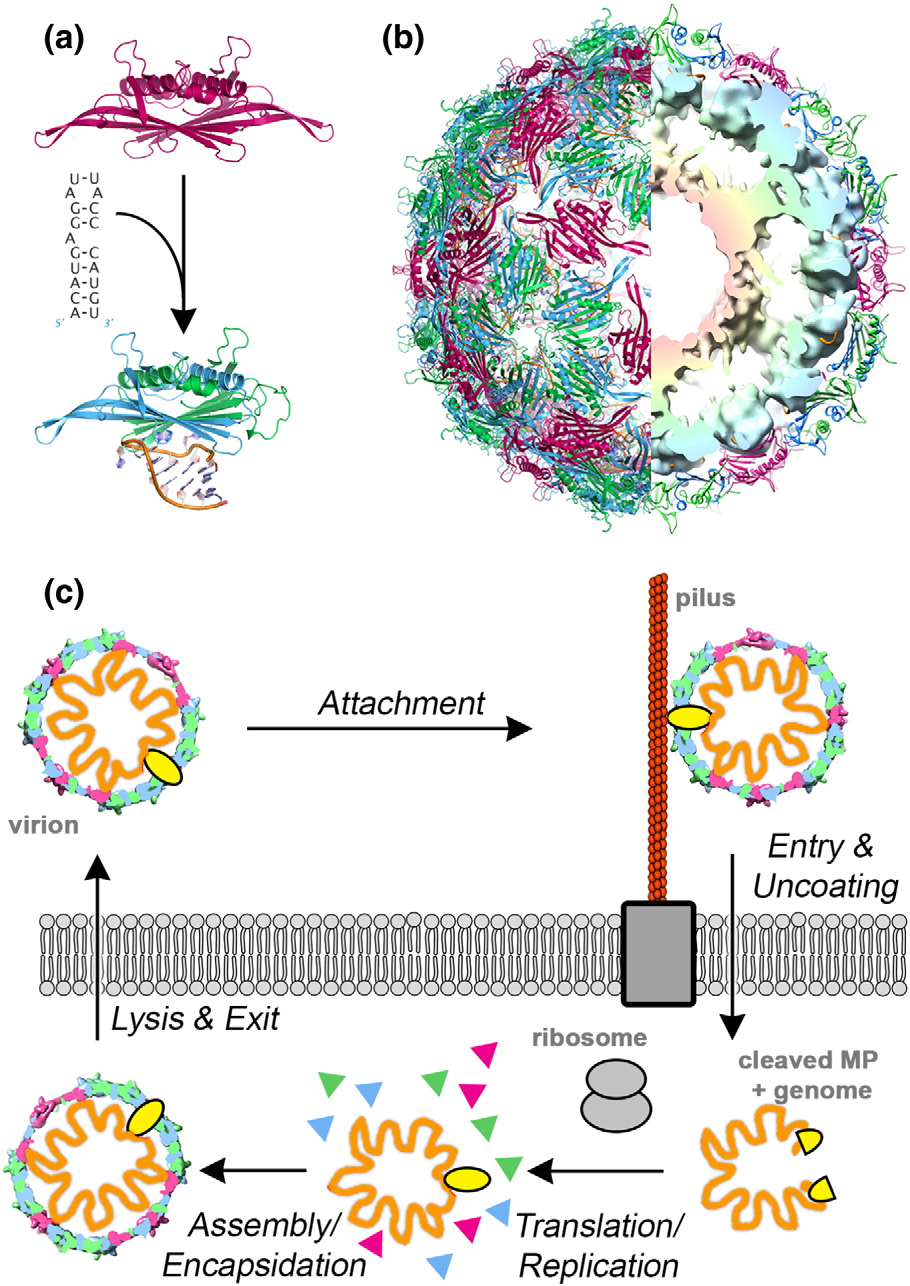
Components of the MS2 virion and its lifecycle. (a) Ribbon representations of the two quasi-equivalent MS2 CP dimers: The symmetric dimer (C/C) is colored pink, while the asymmetric (A/B) is blue/green. Binding the TR high-affinity PS, sequence and secondary structure triggers conformer switching of C/C to A/B [26]. In principle, 60 such conformer switching events are required to create the *T* = 3 capsid shown in (b). (b) Structure of the MS2 virion. Left, surface view of the icosahedrally averaged X-ray structure of the *T* = 3 MS2 virion [27], [28]. Right, cutaway schematic of the equivalent EM reconstitution at 9 Å resolution [29], showing the extensive contacts between the genome, density radially colored pink to blue as the radius increases and the overlying protein shell. X-ray coordinates for these images were taken from PDB ID 1ZDH. (c) Schematic of the phage lifecycle. Virions initially bind to the sides of the bacterial pilus via the MP. MPs are an essential single-copy structural component of RNA phages. By a mechanism that remains largely obscure, the RNA–MP complex but not the remaining capsid shell enters the cell. MP also binds to its own PSs located toward either end of the MS2 genome [30]. Recent asymmetric reconstruction of the MS2–pilus complex suggests that MP replaces a CP dimer of the C/C conformer in an otherwise entirely icosahedral protein shell [31], from which position it is ideally placed to contact the cellular receptor and escort the RNA into the cytoplasm. Note that the presence of the asymmetric MP component could not be detected in averaged X-ray and EM density maps. Once internalized, the MP is cleaved into two separate fragments by protease, allowing translation and replication to start. Temporal control of phage gene expression then regulates the production of progeny genomes and structural proteins that assemble prior to the action of the phage lysis protein.

Most families of these viruses encode single-copy, high-affinity CP-binding sites in their genomes that are thought to act as assembly initiation sites [20], [21]. These RNA sequences form defined secondary structure elements that are specifically recognized by the viral CPs. The best characterized of these interactions is the 19-nt-long stem–loop named TR in the MS2 genome (Fig. 1) that acts as a translational operator for the replicase cistron [22]. This also functions as the assembly initiation site, both *in vitro* and *in vivo* [23], [24]. Mutational disruption of this packaging signal (PS), however, does not result in discernable defects in viral infectivity or virion formation, although similar mutation of the TR recognition site in the CP ablates both these events [25]. This result led to the suggestion that RNA sites other than TR act as the site of assembly initiation in its absence, providing a robust assembly mechanism. Previously, we showed (Fig. 1) that TR–CP interaction has another consequence, namely, that binding of the stem–loop biases the conformational equilibrium of the CP dimer away from the symmetric, RNA-free C/C quasi-conformer and favors the asymmetric A/B dimer [26], [29], [32]. The mechanism underpinning this allosteric conformer switch was investigated using all-atom normal mode analysis [33], [34]. The results suggest that many RNA stem–loops with similar structural features to TR could trigger similar changes; that is, although translational repression is highly sequence specific, the allosteric effect is much less so. This both explains the mutagenesis data and suggests that up to 60 such stem–loops might lie within the MS2 genome, this being the number of A/B quasi-conformer dimers required to construct the *T* = 3 capsid. Electron microscopy (EM) reconstruction of the virion is also consistent with this concept revealing extensively ordered genome segments in contact with the inner surface of the CPs (Fig. 1b, right half). Since the X-ray structures [27], [28], [35] of MS2 and other RNA phages do not show much density for the RNA, the previous inferences based on the apparent absence of such contacts can be also be ignored.

These ideas led to the introduction of a novel model for virion assembly based on the presence of multiple, degenerate and dispersed RNA sequence motifs/structures called PSs that bind their cognate CPs. This concept explains results from single-molecule fluorescence assays of *in vitro* assembly of the model viruses bacteriophage MS2 and STNV [36], [37], [38], [39]. Highly cooperative, selective RNA packaging was observed to occur *in vitro* at nanomolar concentrations, consistent with the existence of multiple dispersed stem–loops in these genomes with recognition motifs similar to the known high-affinity PSs [40], [41]. Mathematical modeling of this mechanism suggests that it provides significant selective advantages for the viruses that use it, including solving the viral equivalent of Levinthal's paradox [42], [43]. Therefore, PS-mediated assembly could be common for viruses that infect many different types of host, although the precise molecular effects arising from PS–CP contacts could be distinct in each case. An essential feature of the PS model is that the dispersed PSs have widely differing CP affinities in order to regulate the number of initiation events occurring and avoid kinetic traps. This implies that PS sequences and structures will be variable and difficult to detect by sequence searches alone. For MS2 (Fig. 1), we have determined the molecular details of sequence-specific recognition of preferred RNA sequences/structures bound by its capsomere [35], [44], [45], [46], [47], [48], [49], [50], [51], [52], [53], [54], a CP dimer (CP_2_), using X-ray crystallography, and we have investigated the PS-mediated assembly mechanism using mass spectrometry [55], analytical ultracentrifugation and smFCS (*s*ingle-*m*olecule *f*luorescence *c*orrelation *s*pectroscopy) [26], [36]. Combined with affinity data [56] and the experimentally determined solution structure of the genome [57], [58], we predicted the sequences and locations of its PSs [41], identifying a large percentage of the putative 60 copies required to regulate assembly of the *T* = 3 shell (Fig. 1).

In order to confirm the presence of multiple PSs within the MS2 genome, we have now determined the contact points between the proteins and RNA components of intact virions. The results show that the previous predictions of PS sites in contact with the phage CP were remarkably accurate, confirming unambiguously the PS-mediated assembly of this virion. The data also allow us to assign the functions of the single-copy/virion maturation protein (MP) to separate domains (Fig. 1), the N-terminal domain seemingly encompassing the RNA-binding function while the pilus-binding function lies within the C-terminal portion. Sequence comparisons with other phage MPs suggest that this is a conserved arrangement. In addition, RNA structure probing has been used to monitor the presence of PS sites in CP-free genome transcripts or the virion, revealing that PSs fall into at least two classes, those that are present at least some of the time in the protein-free RNA and those that must refold as assembly proceeds. An ordered series of induced-fit interactions, similar to those occurring during ribosome assembly [59], [60], [61], [62], likely accounts for MS2 virion formation and presumably for assembly of other ssRNA viruses using the PS-mediated mechanism.

## Results

### Mapping the RNA contacts on both CP and MP subunits

**Fig. 2:**
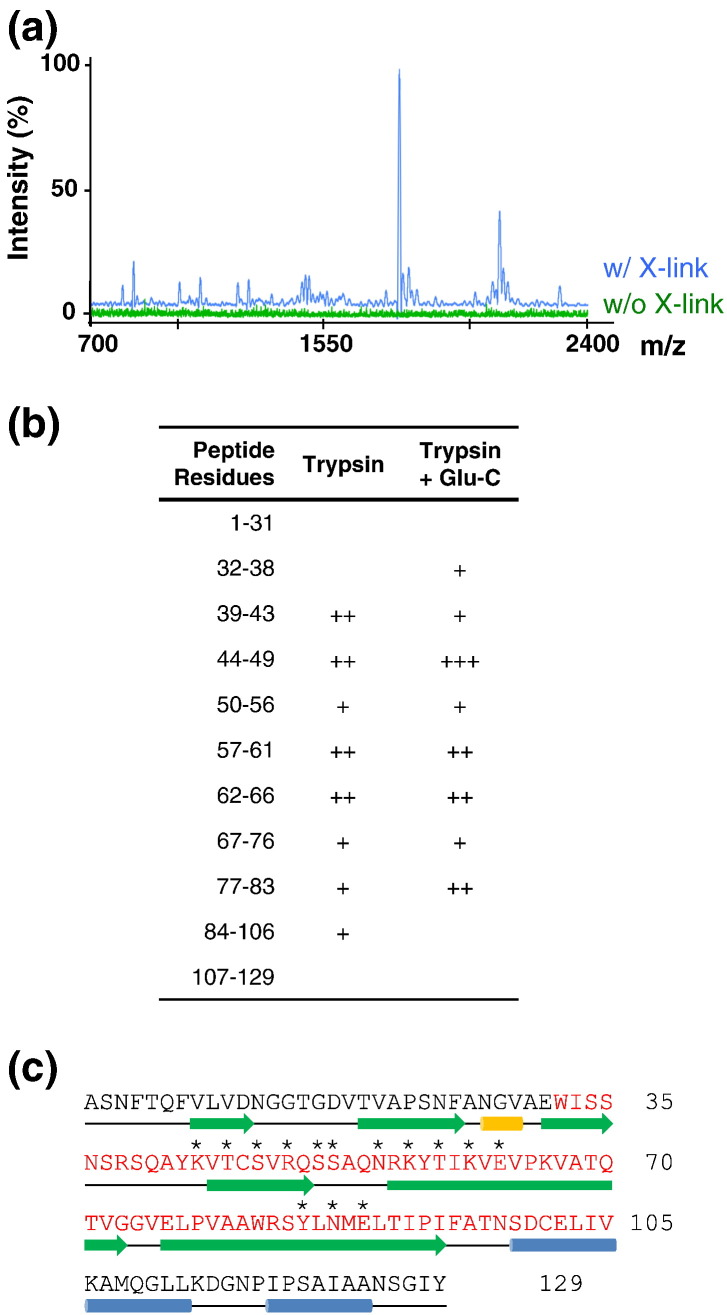
RCAP of the MS2 virion. (a) MALDI-ToF spectra of trypsin-generated peptides co-purified with the MS2 RNA (Materials and Methods) from samples mock-treated or cross-linked with formaldehyde. (b) Residue numbers of CP peptides recovered in experiments similar to those shown in (a) for the different protease treatments. “+” indicates the intensity of each peptide segment. (c) MS2 CP peptides in contact with the genome in the virion. Peptides assigned following RCAP are shown in red and comprise the entire region from amino acid 32 to amino acid 105, which form the four β-strands, C, D, E and F, facing the interior of the virion. The secondary structure elements (PDB ID 2MS2; Fig. 1a) are represented by green arrows, blue bars and a gold bar for β-strands, α-helices and a 3_10_ helix, respectively. Residues contacting the TR PS are shown with an asterisk above (Fig. 1b).

**Fig. 3:**
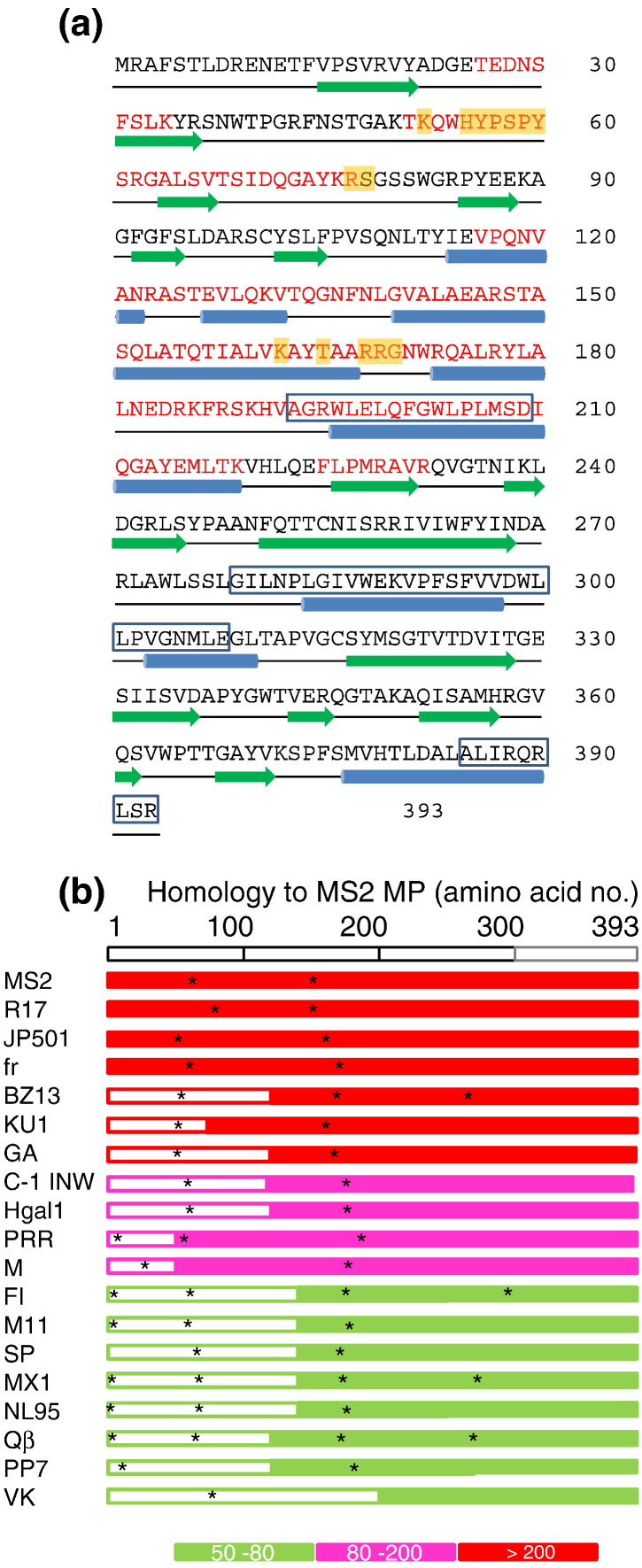
Roles of the MP. (a) Sequence of the MP with the peptides identified via RCAP highlighted in red. The Jpred3-predicted secondary structure elements of the MP are shown using the same format as for the CP (Fig. 2c). Predicted RNA-binding residues (RNABindRPlus) are highlighted in orange. Peptides that are highly conserved across both Leviviridae and Alloleviviridae phage MPs are boxed (motif 1, amino acids 193–210, 51% average identity over 18 amino acids; motif 2, amino acids 279–308, 56% average identity over 30 amino acids; motif 3, amino acids 385–393, average 52% identity over 9 amino acids). These regions may therefore be part of the pilin-binding site. (b) Sequence homology of Leviviridae MP sequences. Filled bars represent regions homologous to MS2 MP and open boxes represent non-homologous regions. These bars cover the full length of each protein, without indicating the short gaps required to accommodate the alignment. Most proteins in this comparison are ~ 400 amino acids long. Colors represent the homology score using the BLOSUM62 matrix. Potential RNA-binding sites predicted by RNABindRPlus are represented by asterisks.

The genomic RNA footprint on the capsid proteins within the virion was determined by an RNA cross-linking and peptide mapping assay (RCAP), used previously to map the interaction between the capsid and the nucleic acid of brome mosaic virus [63]. Briefly, the RNA was cross-linked to the virion proteins in amine-free buffer by treatment with formaldehyde, followed by digestion with proteases. RNA and RNA–peptide complexes were then precipitated with lithium chloride, the cross-links were reversed and the released peptides were identified by MALDI (*m*atrix-*a*ssisted *l*aser *d*esorption/*i*onization)-ToF (*t*ime *o*f *f*light) mass spectrometry (Fig. 2a). Control reactions that lacked formaldehyde did not yield significant amounts of recovered peptides. In contrast, several peptides were detected in the cross-linked samples. Peptides within 0.5 Da of their theoretical molecular mass were assigned to those generated from theoretical digests of the MS2 CP or MP proteins (Fig. 2b and/or c, Fig. 3a, Supplementary Fig. 1 and Supplementary Table 1). There is an extensive overlap in the peptides identified by digestion either with trypsin alone or with trypsin and Glu-C (Fig. 2b). Arginines or lysines that remain uncleaved in these peptides are assumed to be the sites of cross-linking to the RNA.

MS2 virion peptides identified using RCAP are shown mapped onto the amino acid sequences of both the CP (Fig. 2c) and the MP (Fig. 3a). The CP peptides in contact with the genomic RNA encompass all the amino acid residues that contact TR in VLP crystal structures (Supplementary Fig. 1), including the four β-strands (C–F) that form the interior face of the CP subunit. Importantly, there are no RNA contacts that map outside the known RNA-binding site. This is consistent with the idea that CP binding to the genomic RNA occurs along the same RNA-binding face of each CP, as expected for a PS-mediated assembly mechanism.

The RNA–MP complex is the only part of the virion to enter the host cell during infection where the MP undergoes proteolytic cleavage into two fragments of 15 and 24 kDa that are presumed to be separate globular domains [64]. The MP RCAP data (Fig. 3a) are consistent with this idea. All the RNA-contacting peptides are localized to the first 233 amino acid residues of the 393-residue protein, implying that it is orientated in the virion in the C- to N-terminal direction from out to in. This is also consistent with a prediction of RNA-binding functional domains [65], which identifies two motifs located between amino acids 52–60 and amino acids 162–170 (Fig. 3a) that are within peptides detected via RCAP. Sequence comparison with the MPs from other phages reveals a conserved pattern consistent with this organization (Fig. 3b). The MPs of phages in the Levivirus or Allolevivirus families are, respectively, predicted to encode RNA-binding motifs around amino acids 50–70 and 160–180 despite considerable sequence divergence over the N-terminal ~ 140 residues. In contrast, the C-terminal fragments are highly conserved, perhaps reflecting the requirement to bind the same or very similar pilin targets.

### Mapping the CP contacts on the viral genome

**Fig. 4:**
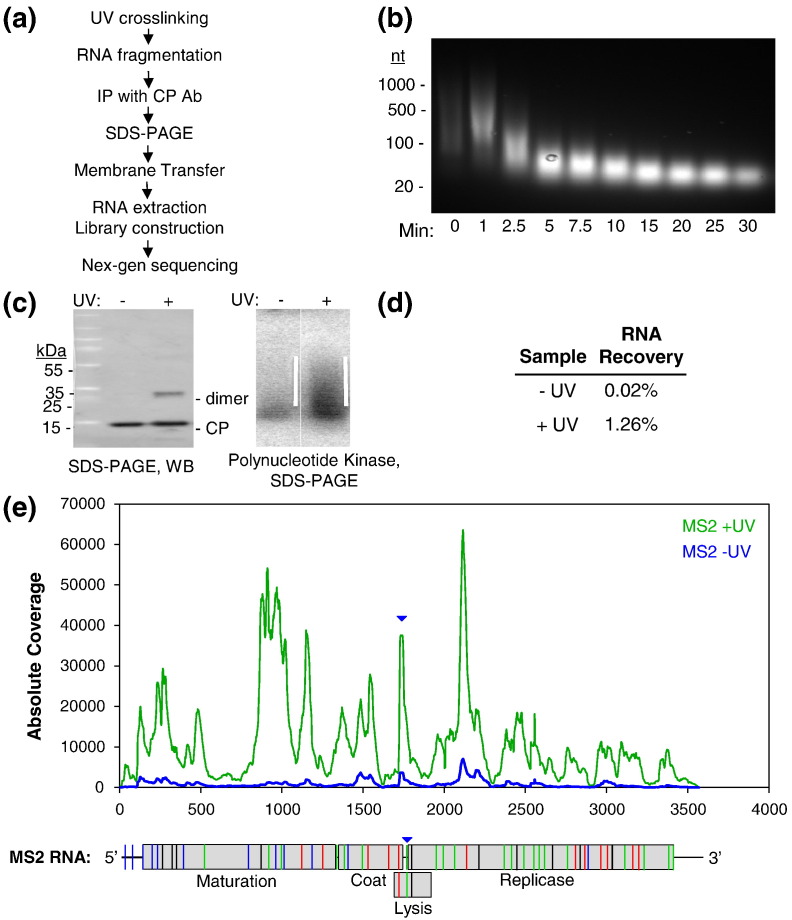
RNA sequences in the MS2 virion that contact the capsid proteins. (a) Outline of the CLIP-Seq protocol used to map RNA residues that contact the MS2 capsid proteins. (b) PAGE analysis of a time course of RNA cleavage using 10 mM ZnCl_2_ with poly(A:U) RNA. (c) SDS-PAGE gel of immune-precipitated MS2 CP containing covalently linked RNA treated with zinc ions. Irradiated and control samples were transferred to a nitrocellulose membrane and subsequently visualized by a Western blot with an anti-MS2 CP primary antibody (left-hand panel). The regions of the same gel containing cross-linked RNA were identified in parallel samples by prior 5′ end labeling of the RNA fragments with polynucleotide kinase (right-hand panel). The smear likely represents cross-linked CP–RNA. The white lines identify the regions excised from the membrane and processed for deep sequencing (see Materials and Methods). (d) Amount of input RNA recovered with CP after purification. (e) Abundances of cDNA fragments that co-purified with the MS2 CP. The sequences for the cDNAs identified by Illumina DNA sequencing were aligned with the MS2 genome, and histograms were produced denoting the frequency of particular sequences within the datasets for irradiated (green) and control (blue) samples. The peak that corresponds to the MS2 operator hairpin is identified by an inverted blue triangle. A schematic of the MS2 genome is shown below the graph to allow identification the approximate locations of the most significant peaks. Lines in blue highlight those sites matching previous PS predictions by Dykeman *et al.*[41]; those in orange, similarly for Bleckley *et al.*[66]; and green, for matches to both predictions. Black lines indicate the 10 peaks not predicted previously.

RNA sequences in contact with the MS2 CP subunits were identified using CLIP-Seq [63]. The genomic RNA was cross-linked to virion proteins using UV irradiation (Fig. 4a). The virion was then dissociated and the protein-bound RNA fragments were minimized by hydrolysis in the presence of zinc ions to produce fragments in the ~ 25- to 40-nt range, based on conditions established for a polyAU control (Fig. 4b). RNA fragments cross-linked to the CP were then concentrated by immunoprecipitation with an anti-CP-specific serum. In the absence of MP-specific antibodies, it is not possible to do the equivalent experiment for RNA fragments in contact with MP. The extent of cross-linking was assessed by SDS-PAGE. UV irradiation produces a small amount of cross-linking between CP dimers, compared to the non-irradiated control (Fig. 4c); however, quantification of radiolabeled RNA revealed that it co-migrated with the CP and that there is an ~ 60-fold higher signal for CP-associated RNA in the irradiated sample (Fig. 4d). The material in these regions of the gel for both irradiated and control samples was then released from the membrane by treatment with proteinase K, TRIzol™ extraction, was ethanol precipitated and was subjected to NextGen sequencing (Illumina MiSeq).

The individual MS2 sequence reads were converted into sense-strand RNA and mapped onto the genomic sequence of the phage creating a histogram of hits at each nucleotide for both irradiated (green) and control (blue) samples, yielding Fig. 4e. The frequency of matched nucleotides varies by factors of up to 6000 across the genome and, at every position, is higher in the cross-linked sample than at the same position in the control. In the sequences from the non-cross-linked sample, some enrichment of the RNAs is apparent. In some places, a small peak in the non-irradiated sample appears to match a peak in the irradiated sample but at a much lower frequency. However, this is clearly not the case for the majority of the peaks in the irradiated sample, confirming that specific enrichment had occurred. The background may be due to some RNA sequences or conformations being recognized by the capsid with higher affinity or that some RNA fragments may precipitate better than others. This is one reason why the peak heights cannot be interpreted simply in terms of RNA–CP affinity.

The pattern of peaks, some of which are clearly symmetrical implying discrete sites of contact, is consistent with the presence of the same series of discrete RNA oligonucleotides in contact with the capsid in every virus particle, that is, that the highest matching nucleotide in each peak should be within or adjacent to a PS. A random arrangement of the genomic RNA within each capsid would not be expected to yield this distribution. Importantly, the level of sequencing multiplicity in the MiSeq data (Supplementary Table 2) suggests that they provide an accurate sampling of the virion population; thus, the peaks represent the most frequently cross-linked fragments. Unfortunately, most peaks are not well resolved from their neighbors, making such direct identification more difficult, but the TR site is clearly within one of these higher peaks (see arrow in Fig. 4e) as expected. In order to analyze these peaks further, we identified a significance threshold (3658; see Materials and Methods) for the number of hits required to generate a significant peak. This results in 54 peaks (Table 1), and their locations are mapped onto the MS2 genome below the histogram of Fig. 4e.

Since cross-linking can occur at any point of contact between a PS fragment and a CP subunit and the rate of zinc ion cleavage is locally RNA sequence/structure dependent, the peak nucleotides do not necessarily coincide with the central nucleotides of a PS. Another problem is the nature of the pile-up analysis could produce a misleading single peak due to overlapping sequence reads from adjoining fragments. In order to take account of these issues, Table 1 lists both the peak nucleotide of each of the 54 peaks and the 20 nucleotides of the genomic flanking sequences on each side of it, thus creating a fragment of about the average probe size that was sequenced. If these fragments encompass PSs, they would be expected to form a stem–loop. Mfold was therefore used to explore the potential secondary structures for each fragment and any stem–loops that can form compared to previous predictions of the PS sites [41]. Sequences capable of forming stem–loops are shown in red in Table 1, with those nucleotides predicted to base pair being underlined. This analysis rapidly identified a large number of potential PSs within the CLIP-Seq fragments. In addition, it revealed several fragments that encompass two potential PSs, presumably for the reasons set out above. It also suggested that the PSs may extend beyond the boundaries of the 41 nucleotides listed, and the regions of these sites within such CLIP-Seq fragments are shown in Table 1. This misalignment could be a consequence of mis-identification of peaks due to their overlaps (Fig. 4e).

There have been two previous attempts to predict the locations of the PSs in the MS2 genome. We used information on the preferred RNA-binding sites for CP from X-ray crystal structures and SELEX together with the reported solution structure of CP-free RNA to create a minimal RNA motif. This yielded 54 of the expected 60 PSs required in this *T* = 3 virus. The CLIP-Seq fragments encompass 33/54 sites predicted in this manner. The Schroeder laboratory has also used their RNA folding algorithm CRUMPLE [66] with a slightly more relaxed probe motif to predict PS sites. The CLIP-Seq dataset has 32 matches to their 60 stem–loop predictions. The matches to the different predictions only partially overlap, meaning that, together, they account for 44/54 experimentally determined sites. Mfold of these remaining 10 orphan sites fails to identify stable folds that are likely PS sites; thus, their presence in the dataset is confusing but could be due to artifacts in aligning CLIP-Seq peaks within the histogram. The multiple matches between the observed CP–RNA contacts in the virion and the predicted PS sites are, however, excellent evidence in support of PS-mediated assembly. It is interesting to examine why neither prediction yielded a complete match to the experimental data, but this requires a more detailed discussion that will be described separately in Dykeman *et al.* (unpublished results).

The CLIP-Seq data also contain a sequence previously reported as one of two RNA-binding sites of the MP, Peak 8 (Table 1) corresponding to the 5´ site. The previous identification of these sites relied on direct sequencing of the RNA fragments resulting from RNase A digestion of the acid-insoluble MP–RNA complex that can be isolated from the virion [30]. This identified two sites, one toward each end of the genome, consistent with a role for the MP in circularizing the RNA. Since the RNA–protein complexes here were recovered by anti-CP antibodies, this suggests that there is a CP-binding site adjacent to the MP-binding site, consistent with the known location of the MP in the virion, where it replaces one of the C/C CP dimers [31].

### Identification of classes of PSs

The experiments described above provide direct evidence that MS2 assembles via a PS-mediated mechanism, but they do not address how the CPs locate their respective PS sites during virion assembly. The latter cannot always be present in the active form of a stem–loop due to the many roles the genome plays in the phage lifecycle. Previous smFCS assays of assembly *in vitro* with the full genome and large sub-fragments, all containing the TR site, show that assembly is divided into two separate kinetic phases. There is an initially rapid phase that occurs above a minimum threshold CP concentration during which the RNA conformational ensemble collapses to facilitate encapsidation [38], [67]. This CP concentration-independent process is followed by a kinetically slower phase, dependent on CP concentration, during which additional CPs are recruited to complete formation of *T* = 3 particles. Controlling the partitioning of these two stages is presumably an essential feature of assembly efficiency. One way that this could be achieved is via PSs with a distinct hierarchy of affinities, with higher-affinity interactions forming in the first phase and lower-affinity ones forming in the later phase. Alternatively, the RNA could undergo conformational change revealing functional PSs after the binding of the initial CP subunits.

**Fig. 5:**
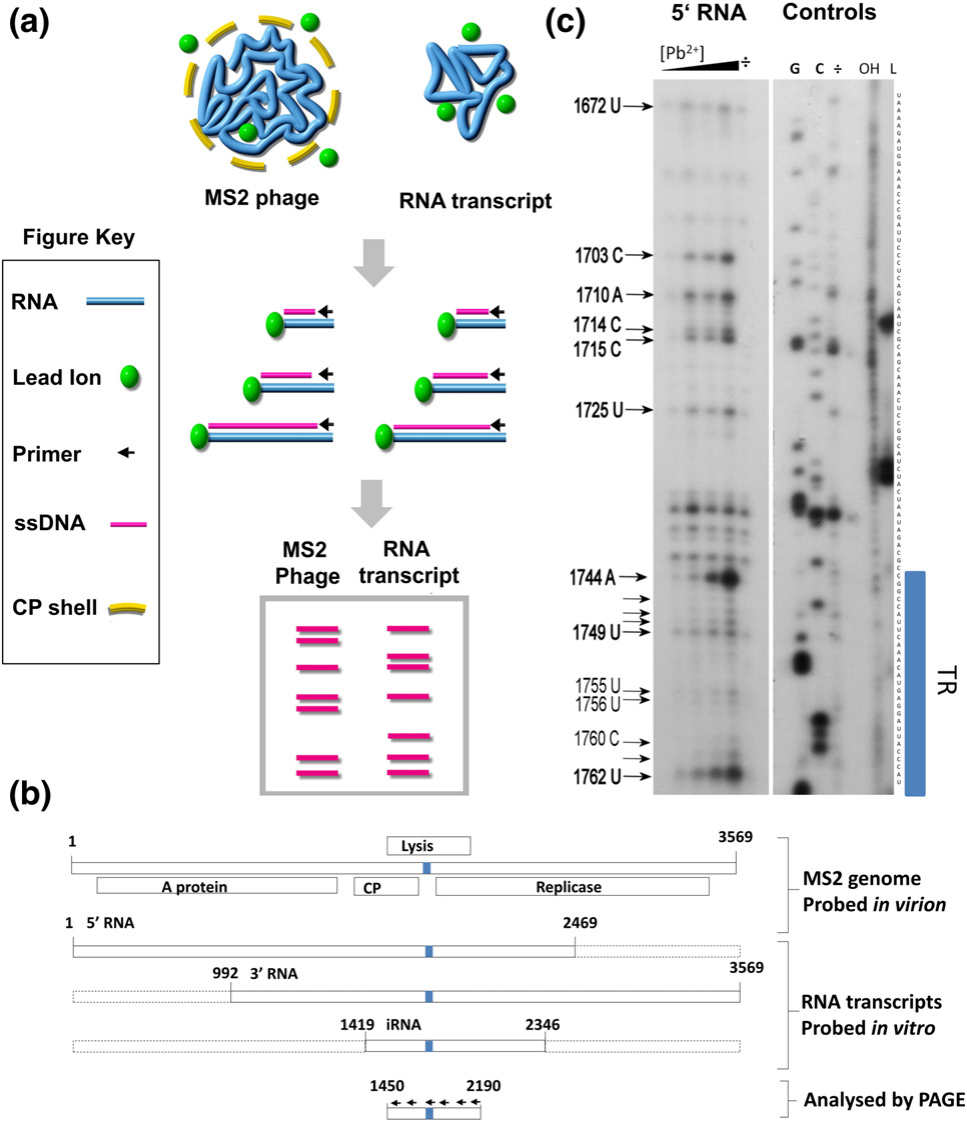
Lead acetate probing of the MS2 ssRNA genome. (a) Outline of the lead probing experiment. The time-dependent cleavage of the genome within MS2 virions or sub-genomic fragments was performed in separate reactions at room temperature. Following Pb^2 +^ incubation, we quenched the reactions with EDTA and we precipitated the RNAs at high salt concentration. Following addition of EDTA, we phenol/chloroform extracted the MS2 phage sample to remove virion proteins. (b) Schematic showing the RNA fragments footprinted and the region from 1419 to 2190 probed. The arrows indicate the sites of hybridisation of the primers; blue bar, the location of the TR site. (c) An autoradiograph of a 6% (w/v) PAGE assay of primer extension products obtained by reverse transcription of the 5′ RNA treated with Pb^2 +^. The TR operator is highlighted by the blue bar. Samples were treated with 0.4 mM Pb^2 +^ in assembly buffer for 5, 10, 30 and 60 min. A control reaction lacking Pb^2 +^ is indicated by “÷”. Arrows highlight sites cleaved specifically by lead-ion-induced hydrolysis positions. Dideoxy sequencing ladders (G and C), a hydrolysis ladder (OH) and size standards (L) analyzed in adjacent lanes allowed identification of the nucleotide sequence and hence the sites of cleavage.

In order to discriminate between these mechanisms, we carried out secondary structure probing of transcripts encompassing large sections of the MS2 RNA, as well as the intact virion (Fig. 5a). This was performed using Pb^2 +^ ions that hydrolyse RNA via the deprotonation of the 2′ OH followed by cleavage of the phosphodiester bond at the 3′ position [68], [69], [70], [71], [72]. Lead ions are ideal for this purpose because their small size allows complete penetration into the MS2 capsids, which have pores at the 3-fold and 5-fold axes, and their reactivity is rapidly controllable by addition of excess ethylenediaminetetraacetic acid (EDTA). In addition, lead ions are expected to have minimal interactions with capsid proteins. The rate of cleavage is affected by the accessibility of the 2′ OH and the flexibility of the RNA backbone. Therefore, efficient cleavage occurs mainly in single-stranded regions and is inhibited in base-paired, stacked or higher-order structures in the RNA or due to interaction with proteins [71].

The lead-ion-induced hydrolysis sites on three MS2 RNA transcripts (referred to as the 5′ RNA, the 3′ RNA and the internal iRNA) were compared to that of the genome within virions (Fig. 5b). We assayed the genome between nucleotide 1450 and 2190, using primers spaced at roughly 130-nt intervals in the MS2 genome, resulting in partial overlap of the primer extension products when resolved on 6–8% (w/v) polyacrylamide sequencing gels. This region is present in all the RNAs, encompasses the TR PS and is well away from MP-binding sites. By analyzing the kinetics of lead-ion-induced hydrolysis, it was possible to assign nucleotide cleavage positions from autoradiographs as signals whose intensities increased with incubation time (see Materials and Methods). An example of the lead-ion-induced cleavage patterns observed on autoradiograms is shown in Fig. 5c. The pattern of cleavages observed within the TR operator in the 5′ RNA is similar in both the 3′ RNA and the iRNA (Supplementary Fig. 2). Three distinct patterns of lead ion cleavage are seen across the different fragments. These are sites that are present in the virion only, in the protein-free RNA fragments only or in both. Note that lead ion cleavage is relatively slow and the signals seen on the gels are from the averaging of many individual molecules. Therefore, it is not possible to be definitive about whether any cleavage sites detected are universally present. However, the qualitative distinction between sites that cleave within the assay period and those that do not provides a clear indication about the propensity for a particular structure to form.

### RNA structure is partially conserved *in vitro* and within the virion

**Fig. 6:**
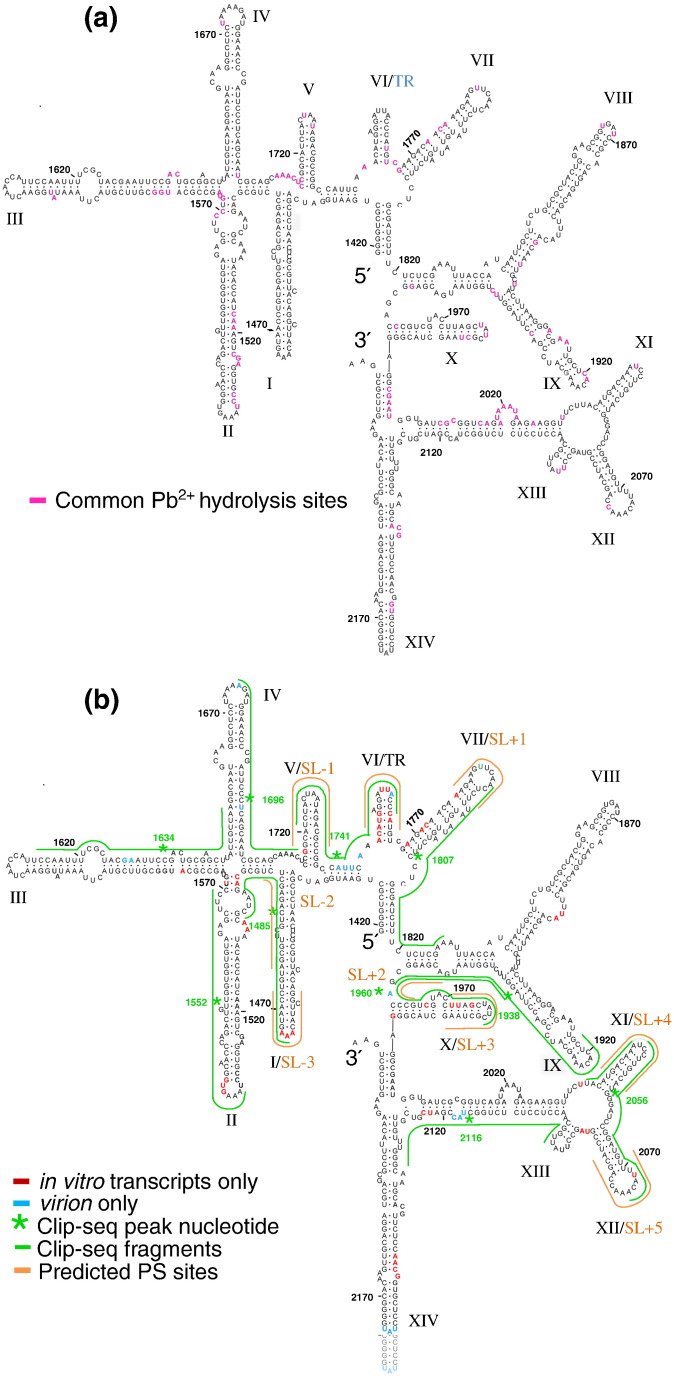
Identification of different classes of PS sites. (a) Shared lead ion cleavage positions detected in both the transcripts and the MS2 virion are highlighted in magenta on the RNA secondary structure [73]. Proposed hairpins are indicated as I–XIV. (b) Altered lead-ion-induced cleavage sites are indicative of altered RNA structure following genome packaging. The lead-ion-induced cleavage sites detected only in transcripts, that is, which are protected in the virion, are highlighted in red. Those that cleave only in the virion are in blue. Comparison of these positions with MiSeq reads, shown here with a green line spanning 40 nt around the peak nucleotide (*), and previously predicted PSs [41] within the MS2 genome, shown here as orange lines and annotated as SL − 3 to SL + 5, allows the identification of PSs that have a propensity to fold prior to capsid assembly or that must refold during assembly.

Conserved lead ion hydrolysis sites were observed across all the RNA fragments that together span the MS2 genome (Supplementary Fig. 2). For example, ~ 75% of the cleavage sites in iRNA are also observed in both the 5′ and 3′ RNAs, with the similarity between the latter two being ~ 90%. These results suggest that the solution secondary structures of the overlapping portions of all three transcripts are very similar. Within the probed region in the virion, there are 121 cleavages, 78 of which (64%) are also detected in each of the protein-free transcripts (Fig. 6a). Most of these are within or adjacent to RNA regions proposed, on the basis of previous selective chemical and enzymatic structure probing, to be single stranded [58], [73]. Thus, some conservation of secondary structures extends to all four RNAs. This is consistent with these features being the result of local interactions, that is, that short-range contacts drive the formation of similar stem–loops [72], [74] that are indifferent to the flanking sequences in each case. For clarity, the stem–loops of the solution secondary structure are labeled 1 to 24 in Roman numerals and, where appropriate, also with our previously predicted PS labels, based on the TR site, that is, SL ± 1 and so on (Fig. 6). Importantly, the clarity of these cleavage patterns suggests that, within each ensemble of probed RNAs, there is a consensus structure and that, within each virion, the genome conformation is largely the same. Such an arrangement is a consequence of PS-mediated assembly and consistent with the structure obtained by asymmetric cryo-tomographic reconstruction [31].

### Identification of the CP–RNA genome interactions in the virion

The conserved solution secondary structure elements in the virion imply that the tertiary RNA conformation is a compacted version of the RNA solution structure [67]. The susceptibility of several nucleotides to lead ion hydrolysis, however, differs between the virion and the protein-free RNA. Some of these differences must be a consequence of the more compact structure, and others likely reflect protection from hydrolysis due to CP binding or a protein-induced remodeling of RNA secondary structure. To identify and differentiate between these scenarios, we compared the oligonucleotides isolated by CLIP-Seq (Fig. 4d and Table 1), the previously predicted PS sites [41], the proposed 2° genome structure [57], [58], [75] and lead ion cleavage positions on protein-free RNA (Fig. 6b).

TR is the highest-affinity PS within the MS2 genome and is thus the site [56] where lead cleavage patterns would be expected to differ the most between solution and the virion. Consistent with this idea, the cleavage patterns of stem–loop VI/TR differ significantly (Fig. 6b) with a large number of nucleotide positions becoming less accessible (red) to the lead ions in the virion, while one nucleotide involved in sequence-specific recognition (Fig. 1b) becomes more accessible (blue). This result suggests that marked alterations in cleavage site accessibility occur as a result of CP binding. A peak centered at nucleotide 1741 in the CLIP-Seq data lies between VI/TR and the 5′ V stem–loop, which we predict is also a PS, SL − 1, confirming the inference from the multiple potential PSs in the CLIP-Seq fragments (Fig. 4d and Table 1) and allowing us to interpret the wider RNA cleavage patterns with confidence.

Starting from the 3′ end of the probed region, there is a CLIP-Seq peak at 2116 that is adjacent to nucleotides with altered accessibility to lead hydrolysis in the virion, implying that this is a PS although it was not previously predicted to be one. The next peak at 2056 lies between two stem–loops predicted to be PSs, XII/SL + 5 and XI/SL + 4, the former having one nucleotide with a detectable change in accessibility. The results with TR and SL − 1 suggest that these sites are indeed PSs. Peak 1960 lies between X/SL + 3 and SL + 2, with both sequences showing some altered lead accessibility, implying two PSs, one present in solution and one that must refold during assembly. Peak 1938 overlaps with the region of SL + 2 and with IX. The latter is not predicted as a PS and shows no changes in lead accessibility; thus, its status cannot be confirmed. Peak 1807 overlaps with VII/SL + 1, which shows some altered accessibility and therefore is likely a PS. Peak 1696 overlaps with V/SL − 1 and IV, the pattern showing increased cleavage at a single nucleotide but seemingly not at a PS. Peak 1634 overlaps an area that undergoes slight changes in accessibility but does not form stem–loops and hence cannot be a PS. Peak 1485 is centered on SL − 2 but is not in the form of a stem–loop and therefore must refold extensively into its PS conformation. It also overlaps with I/SL − 3 where the decreased lead accessibility to the loop region confirms its role as a PS. These correlations with solution secondary structures, CLIP-Seq and PS predictions confirm the ideas of a PS-mediated assembly reaction without the need for extensive mutagenesis that could have pleiotropic effects on MS2 infection.

## Discussion

The virions of ssRNA viruses spontaneously self-assemble *in vitro* and, it is assumed, *in vivo*. This reaction has been proposed to be largely driven by electrostatic interactions. The data presented above show clearly that the end product of such assembly has a highly organized relationship between its virion proteins and genomic RNA, which is most unlikely to arise by a simple charge compaction process. Instead, we have shown that the process is highly regulated and involves multiple RNA–protein contacts at sites distributed throughout the genome, PSs. Such interactions occur in viruses from bacteria (MS2 [2], [23], [31], [37], [41]), plants (TCV [76], BMV [63] and STNV [38]) and humans (HPEV-1 [77]). For BMV, it appears that these interactions can also be altered by post-translational modification of the CP subunits [63] and that virion assembly is controlled by more than electrostatics [78].

PS-mediated assembly sets up a unique relationship between the CP shell and the genomic sequence, allowing the virus to control the spatial positioning of those sequences. This may be an essential feature during the early uncoating events that occur during the infection of the new host since extrusion of the RNA occurs at a unique site in the protein shell [79], [80]. These properties explain why a similar mechanism is found in viruses from such wide-ranging hosts. Since capsid assembly has no cellular homologue that we know of, disrupting PS-mediated assembly can be exploited for therapeutic benefit. Given the potential importance of this mechanism, it is important to validate it as widely as possible. The data presented here contribute significantly to that process. By observing the CP–PS contacts in virions, we have confirmed the roles of multiple PS sites without having to make multiple mutant genomes.

The results are not exhaustive because they do not identify every possible site of contact between virion proteins and the enclosed genome. RNA sequences that lack obvious PS motifs are also present in the CLIP-Seq data, possibly because they contain features that favor precipitation in the CLIP-Seq assay. Nonetheless, the overwhelming majority of contacts are consistent with PS-mediated assembly. The CP peptides in contact with the genomic RNA (RCAP) are similar to those seen in high-resolution X-ray structures of the TR bound to every position. In principle, each PS contact within a genome would be to an A/B dimer (Fig. 1). The VLPs used for X-ray structure determination of bound TR and its variants were soaked with excess oligonucleotide that binds to all dimer sites. At C/C sites, due to the 2-fold symmetry, they make degenerate contacts but bind in a unique orientation at the same RNA–CP interface seen for TR at A/B [27]. The peptide data therefore confirm that every virion has a similar arrangement of RNA–protein contacts for the first time. In addition, CLIP-Seq has been used to identify the RNA sequences in close contact with these peptides, revealing extensive overlap with previous predictions of PSs within this virus. This is the most compelling evidence to date confirming that MS2 assembly occurs via multiple PS–CP contacts.

We have also investigated the architecture of the MP. Its contacts to the genome are localized to the N-terminal half of the molecule, consistent with predictions of two RNA-binding sites in this region. Although these RNA-binding motifs seem to occur across a large range of the known MPs, the sequences of these N-termini are quite variable. In contrast, there is extensive conservation at the C-termini, perhaps consistent with it encompassing the pilus-binding domain.

Combining CLIP-Seq information with RNA chemical structure probing allows us to interrogate the presence/appearance of PSs before and after virion assembly. It is clear that there are two classes of PS sites, those that are detectable in the protein-free RNA and those that are only detected after CP-induced refolding and capsid assembly. Presumably, the PS folds are flickering in and out of existence and the lead ions are sensitive to equilibrium. This is consistent with the two phases of assembly previously detected using smFCS *in vitro*. This scenario is somewhat analogous to that seen for assembly of the ribosome [59], [60], [61], [62]. However, ssRNA viruses face a more complex assembly task than the ribosome. They must co-assemble around RNA genomes, often many times longer than any ribosomal RNA, using multiple copies, often numbering in the hundreds, of the same or very similar CP subunits, to form a highly symmetrical container of restricted volume. Confining a complex folded polymer, such as genomic RNA, into the limited volume of the viral capsid requires proper regulation. Furthermore, the assembly of the virion must allow proper extrusion of the RNA and proper viral gene expression in the subsequent round of infection. Thus, while electrostatic interactions are useful in collapsing the conformational ensemble of protein-free RNA and for lowering the overall free energy of the assembled particle, rapid and efficient assembly requires that they occur in a regulated fashion. This control is precisely what PS-mediated assembly provides. The tendency of viral genomes to form branched structures can also contribute to this assembly efficiency [81], [82]. Since PS binding by MS2 CP leads to conformational switching in the CP dimer and CP binding to PSs results in local refolding of the genome, assembly occurs via a series of events that each relies on induced fit. Given that this is a highly cooperative event involving multiple RNA–protein and protein–protein contacts, the free energy involved in this induced fit is likely to be considerable. This idea is not new [83], but here we have been able to probe the details of how the process is controlled by the genomic PSs for the first time.

## Materials and Methods

### Defining the RNA–protein contacts in the virion

#### RCAP

MS2 virions were from the American Type Culture Collection and propagated in male strains of *Escherichia coli*. The virions were purified [84] and suspended in SMH buffer [100 mM NaCl, 8 mM MgSO_4_ and 50 mM Hepes (pH 7.5)] to identify regions in the MS2 CP and MP that contact the RNA. The RCAP protocol was as described in Ni *et al.* [63]. Aliquots of the trypsin-treated samples were subsequently treated with sequencing-grade Glu-C (Promega) prior to purification, concentration of the sample using a c18 ZipTip and mass spectrometric analysis using a Bruker Autoflex III mass spectrometer (Agilent Technologies) in positive-ion mode. Some samples were also examined using the reflectron mode. Database searches used the program Mascot (Matrix Science), with the search directed against the MS2 protein sequences [53]. Peptide assignments were only made when they were within 0.5 Da of the expected mass.

#### CLIP-Seq

MS2 virions in SM buffer [100 mM NaCl, 8 mM MgSO_4_ and 50 mM Tris–HCl (pH 7.5)] were irradiated at 400 mJ/cm^2^ by 254-nm light, on ice in a CL-1000 ultraviolet cross-linker (UVP). The samples were mixed with gentle pipetting and incubated on ice for 3 min. Irradiation was repeated twice more. The virions were then pelleted by ultracentrifugation at 60,000 RPM for 4 min at 4 °C and resuspended in RNA fragmentation buffer [100 mM Tris–HCl (pH 7.0) and 10 mM ZnCl_2_] at 70 °C for 20 min. The reaction was quenched by adding EDTA, pH 8, to a final concentration of 42 mM. Immunoprecipitation used an anti-MS2 CP serum (EMD Millipore) bound to protein A/G magnetic beads (Pierce) according to the manufacturer's protocol. The eluted CP–RNA complex was electrophoresed on a NuPAGE 4–12% (w/v) Bis-Tris SDS-PAGE gel in 1 × 4-morpholineethanesulfonic acid buffer on ice and then transferred to a nitrocellulose membrane using a wet transfer apparatus (BioRad) at 100 V for 1 h. The membrane was stained with 0.1% (w/v) Ponceau-S in 5% (v/v) acetic acid for ~ 5 min, and the region 6–100 kDa above the CP band was excised from the membrane. Material on the membrane was digested with 0.8 mg/mL proteinase K at 37 °C for 1 h to release the cross-linked RNA fragments. The RNA was extracted using Trizol and precipitated with 3 volumes of ethanol containing 0.3 M ammonium acetate.

In preparation for cDNA library construction, the RNA was treated with T4 polynucleotide kinase (NEB) to remove the 3´ phosphate and add a 5´ phosphate. The pre-treated RNA was then prepared using an Illumina Truseq Small RNA preparation kit. After PCR amplification of the cDNA using Pfu DNA polymerase, the reaction was incubated with 1 μL Taq DNA polymerase at 72 °C for 10 min and the amplified cDNA was purified with a Qiaquick Gel Extraction Kit (Qiagen) from a 2% (w/v) agarose-TAE gel. The libraries from different samples were pooled at equimolar ratios for Illumina MiSeq sequencing using MiSeq reagent kit V3 (150 cycles). All samples from the UV cross-linked and un-cross-linked samples were processed in parallel and sequenced within the same run.

#### Labeling of CP-bound RNA

To visualize and quantify the RNAs covalently attached to MS2 CP, we incubated CP–RNA complexes treated with ZnCl_2_ with 2 U of T4 polynucleotide kinase and [γ-^32^P]ATP for 30 min at 37 °C according to the manufacturer's instructions (NEB). The material was then electrophoresed in NuPAGE 4–12% (w/v) Bis-Tris SDS-PAGE gel in 1 × 4-morpholineethanesulfonic acid buffer. The gel was wrapped in plastic film and the signal was detected using a Phosphorimager. Western blots of the CP were performed as described in Hema *et al.* [85].

#### DNA analysis

Analysis of the MiSeq reads used the Galaxy user interface [86]. The reads were processed with the FASTX toolkits to trim sequences with a quality score lower than 20 using *FASTQ Quality Trimmer,* removing the 3´ adaptor and discarding any reads shorter than 15 nt using *Clip*. The cleaned reads were aligned to the MS2 reference genome using Bowtie2 [87] under the default “local” alignment settings and the actual coverage of each nucleotide position on MS2 RNA was obtained using Mpileup in SAMtools [88]. The Mpileup output from the forward and reverse MiSeq reads were combined. All peaks with nucleotide coverage higher than the threshold of 3685 reads were assigned as putative capsid-interacting sites. The threshold was established with results from a “no UV” control library, using the number of reads at the apex of the translational operator (TR) hairpin as the cutoff.

#### MP sequence analysis

*Leviviridae* MP sequences were identified using BLASTP and TBLASTN searches at National Center for Biotechnology Information [89] using MS2 MP as the query (sequence accession P03610). Excluding MS2 search results and using an Expect value of 10 allowed recovery of low homology sequences. Matches were scored using default parameters, with the BLOSUM62 matrix.

MS2 MP secondary structure composition was predicted using the Jpred3 algorithm [90] with sequence accession P03610 query and homologous UniRef sequences identified by the server. CP secondary structures were taken from the annotation of PDB file 2MS2.

RNA-binding sites in MPs were predicted using the RNABindRPlus server [65] with sequence accessions MS2 (P03610), R17 (EF108465), JP501 (AAF67668), phage fr (CAA33135), BZ13 (FJ483837), KU1 (AAF67673), GA (CAA27496), C-1-INW (AFN37812), Hgal1 (AFN37816), PRR (ABH03626), phage M (JX625144), phage FI (ACT66757), M11 (AAC06249), phage SP (X07489), MX1 (AF059242), NL95 (AF059243), Qβ (AY099114), PP7 (A80191), VK (EU372698) and AP205 (AF334111).

### Probing RNA conformation before and after encapsidation

#### Lead ion probing

The production and purification of the MS2 RNA fragments was as previously described [67]. All structural probing reactions were performed in 10 μL final volume. The 5´ and 3´ sub-genomic fragments were at a 0.1 μM final RNA concentration. The iRNA was kept at 0.27 μM final concentration. RNA concentrations were measured by absorbance at 260 nm assuming that one asymmetric unit is equivalent to 40 μg/μL. Wild-type MS2 phage was used at a final concentration of 0.285 μg/μL. Prior to cleavage with lead ions, the MS2 sub-genomic RNAs were heated to 65 °C for 10 min and then cooled to room temperature at 2 °C/min on a thermal cycler in 6 μL final volume. At this point, 2 μL of 5 × assembly buffer was added to the sample resulting in a final concentration of 40 mM NH_4_OAc and 1 mM Mg(OAc)_2_ (pH 7.2). We then added 2 μL of freshly prepared 2 mM lead acetate to each reaction to a final concentration of 0.4 mM lead ions. Reactions were incubated at room temperature for 5, 10, 30 and 60 min followed by addition of 5 μL of 0.1 M EDTA, 1.5 μL of 3 M NaOAc and 35 μL of EtOH and were stored at − 20 °C for ≥ 2 h. RNA was recovered as a pellet by centrifugation at 13,000 RPM and washed once with 70% EtOH prior to resuspension in 5 μL DEPC-treated H_2_O. Wild-type MS2 capsids were treated similarly but were phenol/chloroform extracted prior to precipitation with 3 M NaOAc. Prior to reverse transcription, the appropriate ^32^P-labeled primer was annealed to the cleaved RNA by addition of 1 μL of 1 μM labeled primer stock solution and incubated at 65 °C for 10 min followed by snap cooling on ice. The primers used for reverse transcription were as follows:

1568_R:AAGCTCTACACCACCAACAGTCT
1666_R:CCTTGCATTGCCTTAACA
1812_R:CGCGAGGAAGATCAATACATA
1951_R:TCATTACCAGAACCTAAGGTCGGA
2061_R:GATCCCATGACAAGGATTTG
2223_R:TAACGGTTGCTTGTTCAGC

Reverse transcription was performed with Transcriptor® reverse transcriptase (Roche Diagnostics) at 52 °C for 30 min according to the manufacturer's description. Single-stranded DNA products were then precipitated by addition of 3 μL of 3 M NaOAc and 60 μL of EtOH, recovered by centrifugation and washed once with 70% EtOH and allowed to dry at room temperature for 15 min. Single-stranded DNA was dissolved in 95% formamide RNA loading buffer and run on 6–8% (w/v) denaturing polyacrylamide slab gels [91]. The gels were then exposed to film at − 80 °C overnight and digitized using an image scanner.

#### Data analysis

Lead ion cleavage positions were identified from exposed film first and foremost as signals whose intensity increased with increasing lead ion incubation time. An absence of a signal in the control lane was taken as an indicator but not a requirement of lead-ion-induced cleavage. Because control reactions were incubated in assembly buffer for 60 min at room temperature, non-specific hydrolysis sites were always detected. If intensity at these positions was observed to increase in the presence of lead acetate over time and the intensity with lead ions *versus* control after 60 min was higher in sample containing lead ions, this was defined as a hydrolysis position. The nucleotide position of induced hydrolysis within the MS2 genome was deduced from dideoxy sequencing ladders (G and C) and a size standard. Identified positions were not quantified, and thus, a comparison of cleavage intensities between RNAs was not performed. All lead-ion-induced hydrolysis was therefore identified in a Boolean manner with regard to the abovementioned criteria. Lead ion hydrolysis positions were mapped onto the MS2 genome and their nucleotide positions were compared with respect to whether cleavage occurred only in the RNA sub-genomic fragments *in vitro*, only within the virion or in both the virion and the sub-genomic fragments *in vitro*. The numbers of cleavages occurring in these three groups were taken as an indicator of structural similarity of these RNAs in the different environments.

The following are the supplementary data related to this article.

Supplementary Tables

Supplementary Fig. 1 Peptides in contact with genomic RNA in the virion. Peptides from (a) the CP or (b) MP identified by MALDI-ToF of formaldehyde cross-linked virions are highlighted by colored bars above the sequences for trypsin digestion alone (red) or trypsin plus Glu-C (blue). These results are also summarized in Fig. 2 and Supplementary Table 1. (c) Details of the TR–CP A/B dimer interaction seen in VLPs containing multiple copies of TR [35]. Nucleotide numbering is relative to the first nucleotide of the replicase start codon (A_− 1_). Protein–RNA contacts seen in the X-ray crystal structure are shown as dotted lines. Peptides cross-linking to the genomic RNA are in red (see Fig. 2b).

Supplementary Fig. 2 Comparison of lead ion cleavage maps. The map highlights all lead ion cleavage positions identified within the 1450-2190 region of all sub-genomic RNAs and the genomic RNA within the virion. Cleavage sites are indicated with vertical bars that are color coded according to the RNA. No discrimination is made between strong and weak cleavage positions when the cleavage sites were identified from autoradiographs. The annealing positions of the primers used in primer extension reactions are shown as black arrows.

## Figures and Tables

**Table 1:**
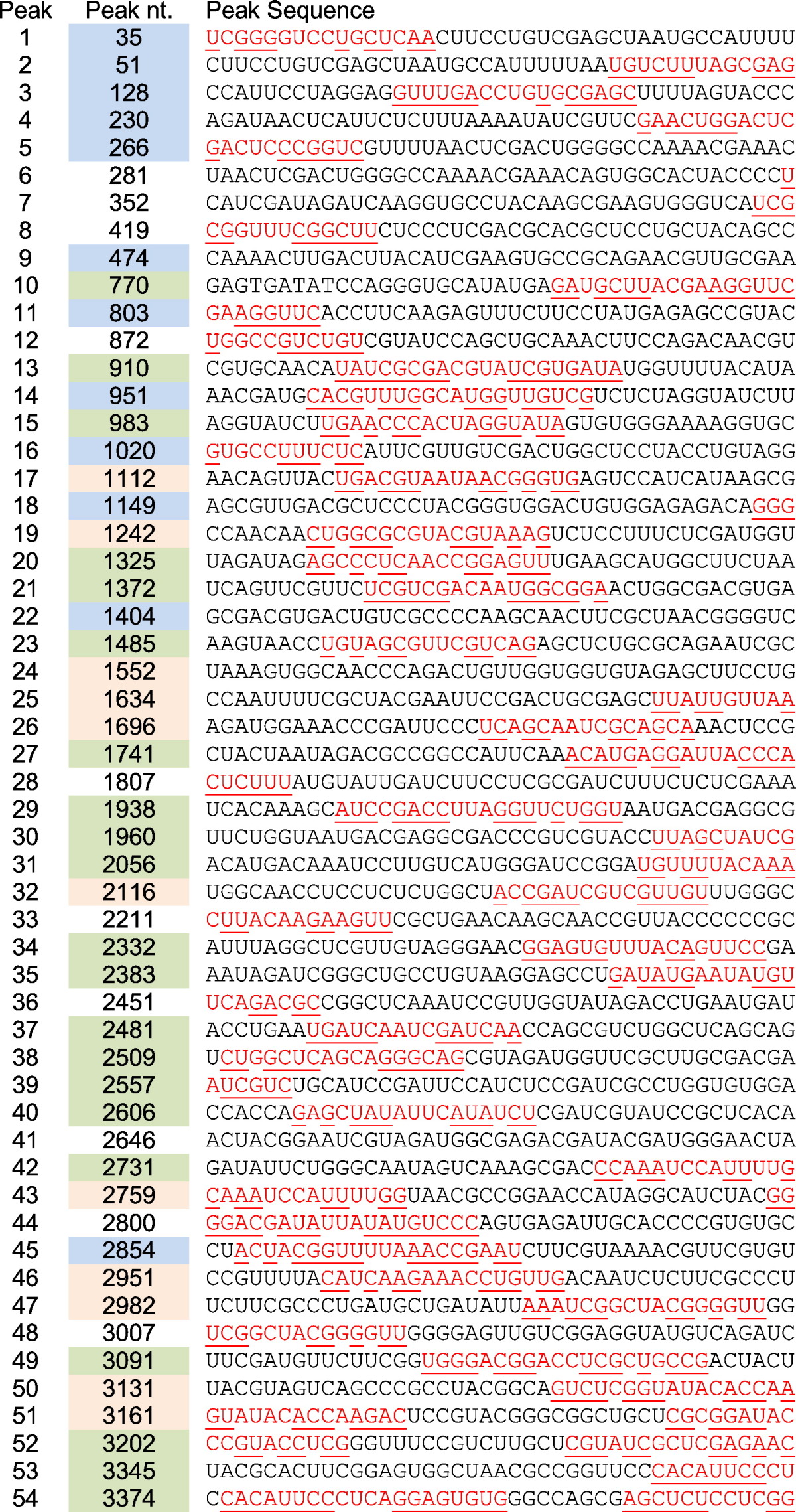
Flanking sequences of the MiSeq peaks and their relationship to previous predicted PSs. Matches to the predictions of Dykeman *et al.*[41] are highlighted in blue in the peak nucleotide. Column, those to Bleckley *et al.*[66], is in orange and with matches to both in green. There are 10 CLIP-Seq sites that do not match any prediction (no color). Predicted hairpins are underlined. Predicted MS2 CP-binding motifs are in red. The peak column relates to the peaks identified in Fig. 4e with peak 1 nearest the 5′ end of the genome.
