# Supplementary material for: Direct Evidence for Packaging Signal-Mediated Assembly of Bacteriophage MS2
Source: J Mol Biol. 2016 Jan 29;428(2):431–48. doi: 10.1016/j.jmb.2015.11.014 (PMC4751978; doi:10.1016/j.jmb.2015.11.014)
Supplement: Supplementary file 1 — Supplementary Tables [file mmc1.pdf]

SUPPLEMENTARY RESULTS

Supplementary Table 1. Summary of peptides derived from the MS2 MP that were cross-linked to the MS2 genomic RNA.

Peptide Residues	Trypsin	Trypsin + Glu-C
1-25		
26-34		+
35-50		
51-52	+	
53-62	++	+
63-77	+	
78-115		
116-131		+
132-162	+	+
163-169	+	+
170-177	++	++
178-184	+	+
185-190	+	++
191-198		++
199-209		+
210-219		+
220-224		
225-232		+
233-243		
244-260		
260-393		

Supplementary Table 2. Summary of data from Illumina DNA sequencing of cDNAs derived from RNAs bound to the MS2 CP.

Library	Mapped Reads F.	Average Coverage	Mapped Reads R.	Average Coverage R.	Total Mapped Reads	Total Avg. Coverage
MS2, naked RNA	540,942	3834	607,844	9,028	1,148,786	12862
MS2, UV 0x CP	29,157	211	30,293	645	59,450	856
MS2, UV 3x CP	390,988	2820	440,087	7843	831,075	10667

Mapped reads F/R define the number of forward/reverse reads, respectively, present in the DNA sequencing run that corresponded to MS2 genome. “Coverage” is the number of sequence matches to the MS2 genome in the NextGen data.
