# Supplementary figures and images for: Direct Evidence for Packaging Signal-Mediated Assembly of Bacteriophage MS2
Source: J Mol Biol. 2016 Jan 29;428(2):431–48. doi: 10.1016/j.jmb.2015.11.014 (PMC4751978; doi:10.1016/j.jmb.2015.11.014)

## Slide 1
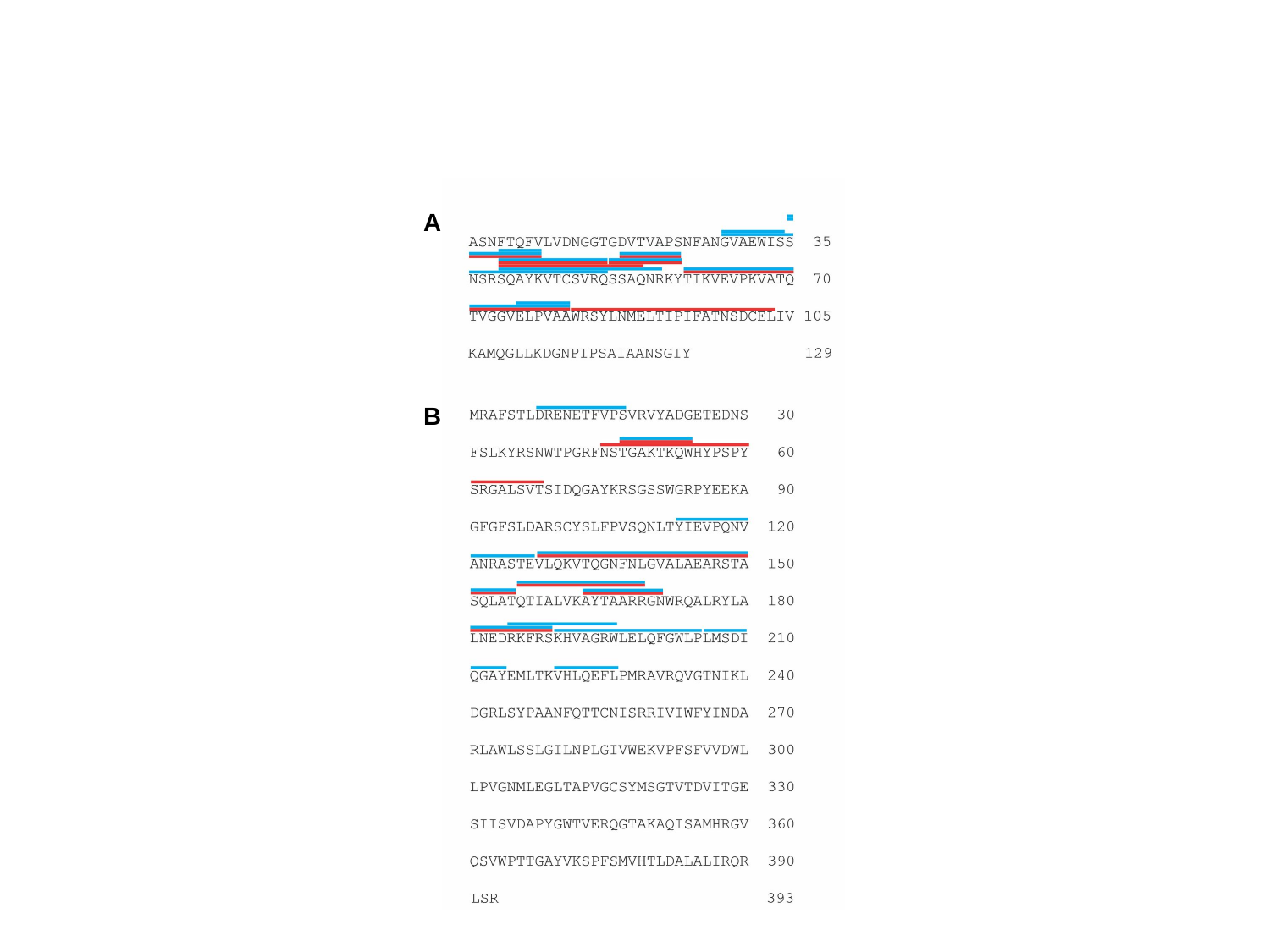

A
B

Supplement: Supplementary Fig. 1 — Peptides in contact with genomic RNA in the virion. Peptides from (a) the CP or (b) MP identified by MALDI-ToF of formaldehyde cross-linked virions are highlighted by colored bars above the sequences for trypsin digestion alone (red) or trypsin plus Glu-C (blue). These results are also summarized in Fig. 2 and Supplementary Table 1. (c) Details of the TR–CP A/B dimer interaction seen in VLPs containing multiple copies of TR [35]. Nucleotide numbering is relative to the first nucleotide of the replicase start codon (A− 1). Protein–RNA contacts seen in the X-ray crystal structure are shown as dotted lines. Peptides cross-linking to the genomic RNA are in red (see Fig. 2b). [file mmc2.pptx]

## Slide 1
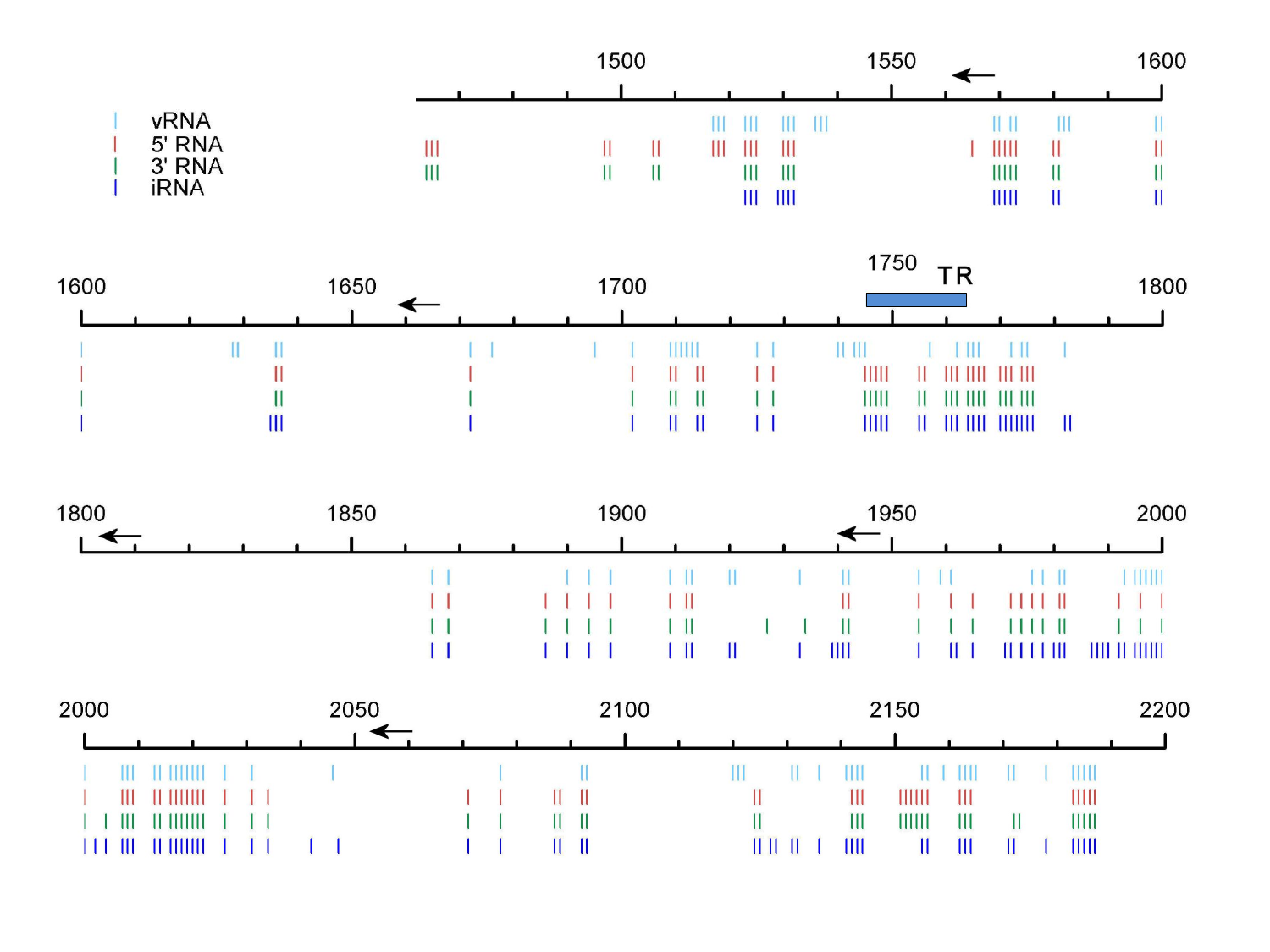

Supplement: Supplementary Fig. 2 — Comparison of lead ion cleavage maps. The map highlights all lead ion cleavage positions identified within the 1450-2190 region of all sub-genomic RNAs and the genomic RNA within the virion. Cleavage sites are indicated with vertical bars that are color coded according to the RNA. No discrimination is made between strong and weak cleavage positions when the cleavage sites were identified from autoradiographs. The annealing positions of the primers used in primer extension reactions are shown as black arrows. [file mmc3.pptx]
